# Aromatic compounds analysis of Guizhou honey-sweet tobacco

**DOI:** 10.3389/fpls.2026.1878964

**Published:** 2026-07-15

**Authors:** Yuge Zhao, Huifei Li, Risheng Zhong, Liang Feng, Shuqi Wang, Yechun Lin, Haitao Chen

**Affiliations:** 1 Beijing Advanced Innovation Center for Food Nutrition and Human Health, Beijing Key Laboratory of Flavor Chemistry, Beijing Technology and Business University (BTBU), Beijing, China; 2 Key Laboratory of Geriatric Nutrition and Health (Beijing Technology and Business University), Ministry of Education, Beijing, China; 3 Guizhou Academy of Tobacco Science, Guiyang, Guizhou, China

**Keywords:** aroma extract dilution analysis, aroma-active compounds, flavor, honey-sweet flue-cured tobacco, volatile

## Abstract

Guizhou honey‑sweet type flue‑cured tobacco is of high commercial value, but the key aroma‑active compounds responsible for its characteristic flavor have not been systematically validated at the sensory level. To address this gap, we employed molecular sensory science approaches, including aroma extract dilution analysis (AEDA), quantitative gas chromatography–mass spectrometry (GC–MS), odor activity value (OAV) calculations, sensory evaluation, and aroma recombination/deletion experiments, on grade B and C leaves from Zunyi (ZY), Tongren (TR), and Hubei (HB). A total of 42 aroma‑active compounds were detected, of which 23 were confirmed. β‑Damascenone and phenethyl alcohol constituted the common core aromatic skeleton across samples, while ZY‑C exhibited the richest composition with 10–12 key compounds (OAV ≥ 1). Sensory evaluation results were consistent with OAV findings, with ZY‑C showing the most favorable overall aroma profile. Recombination experiments successfully reproduced fruity and floral notes but failed to adequately replicate roasted and hay‑like aromas. Deletion experiments confirmed that acetic acid, β‑damascenone, neophytadiene, phenylacetic acid, and maltol significantly contributed to the overall aroma, with phenylacetic acid and maltol identified as the key chemical basis for the honey‑sweet characteristic of HB‑C. This study provides a scientific foundation for the targeted regulation of tobacco leaf flavor.

## 1 Introduction

Tobacco originated in the Americas and has been cultivated in China for over 400 years since its introduction in the 16th century; it is now widely grown in tropical to temperate regions worldwide. China is the world’s largest producer and consumer of tobacco, with Yunnan, Guizhou, and Henan among the primary growing regions (Zhu, 2009). Flue-cured tobacco, prized for its mellow aroma and smooth taste, dominates China’s cigarette industry. In the early 21st century, the State Tobacco Monopoly Administration proposed the strategic goal of “high aroma, low tar, and low harm.” However, tar-reduction measures often result in a simultaneous reduction of nicotine and aroma-contributing compounds, thereby compromising the richness of cigarette aroma. Consequently, industry strategies must shift from a sole focus on tar reduction to a balanced approach that prioritizes both harm reduction and aroma preservation (Zhu, 2006). There is an urgent need to develop tobacco varieties that possess ample aroma while maintaining appropriate levels of nicotine and tar. Tobacco leaf quality is regulated by the synergistic interaction of multiple factors, including genetics, ecology, and cultivation, and its chemical composition is highly complex. A thorough analysis of the chemical basis underlying its aroma profile is currently the central focus of tobacco flavor research (Cao, 2022).

As a major production area for high-quality flue-cured tobacco in China, Guizhou benefits from its unique plateau climate and yellow soil conditions. The tobacco leaves produced there exhibit a distinctive “honey-sweet aroma” profile, playing an irreplaceable role in flavor profiling and quality enhancement in cigarette formulations. Previous studies have systematically identified the characteristic volatile organic compounds (VOCs) of honey-sweet tobacco leaves and preliminarily clarified the contributions of various aromatic components to their flavor profile (Zhong et al., 2025). However, existing studies have primarily focused on the qualitative identification and comparative analysis of compound concentrations, with a lack of systematic quantitative validation and sensory confirmation regarding the specific contributions of key aroma-active components within the overall flavor system and their interactions (Zhong et al., 2025).

However, existing studies have several limitations. Most have focused on qualitative identification and comparative analysis of volatile organic compound (VOC) concentrations, without systematic quantitative validation of the specific contributions of individual aroma-active components. Furthermore, the sensory interactions among these compounds and their collective role in producing the characteristic honey-sweet aroma remain unexplored. Critically, no study has employed molecular sensory science methods—such as aroma recombination and omission experiments—to confirm the sensory relevance of key aroma compounds in Guizhou honey-sweet tobacco. Therefore, the present study was undertaken to fill this knowledge gap. Specifically, we selected four typical Guizhou honey-sweet flue-cured tobacco samples (ZY-C, ZY-B, TR-C, HB-C) and applied a comprehensive molecular sensory approach comprising solvent-assisted flavor extraction, GC-O-MS with AEDA, quantitative GC-MS, OAV calculation, sensory evaluation, and aroma reconstruction/depletion experiments. This work advances current knowledge by: (1) providing the first systematic sensory validation of key aroma-active compounds in this tobacco type; (2) quantifying the relative contribution of each compound via OAV and FD factors; and (3) experimentally confirming the indispensable role of selected compounds through omission testing. The findings elucidate the chemical basis of the honey-sweet aroma and offer a scientific foundation for quality evaluation and targeted flavor regulation.

## 2 Materials and methods

### 2.1 Tobacco samples

Four flue-cured tobacco leaf samples representing different geographic origins and grades were investigated: the middle portion of tobacco leaves from the Zunyi region (ZY-C), the upper portion of tobacco leaves from the Zunyi region (ZY-B), the middle portion of tobacco leaves from the Tongren region (TR-C), and the middle portion of tobacco leaves from the Hubei region (HB-C). All samples were harvested in autumn 2023 from plants grown for 110–120 days after transplantation. All samples were provided by the Guizhou Tobacco Corporation. Their grades were evaluated by an expert panel organized by the Zhengzhou Tobacco Research Institute, comprising appearance quality experts from China Tobacco Hunan and Guizhou Provincial Tobacco Bureau, as well as members of the National Tobacco Standardization Subcommittee, senior grading technicians, and national grading competition champions. Each tobacco sample was analyzed using three independent biological replicates (n = 3), with each replicate consisting of a separate pool of leaves from five randomly selected plants grown under identical conditions.

### 2.2 Chemicals and reagents

Dichloromethane (HPLC grade) was purchased from Thermo Fisher Scientific (USA). Deuterated n-hexanol (D13, 0.948 mg/mL) was supplied by Sigma-Aldrich (USA) and used as an internal standard. Anhydrous sodium sulfate (analytical grade) was purchased from China National Pharmaceutical Group Chemical Reagents Co., Ltd. (China). Standard samples of aromatic compounds were purchased from Sigma-Aldrich (USA), Alfa Aesar (USA) and J&K Scientific (China). Odorless cellulose powder (particle size ≤ 25 μm) was supplied by Aladdin and used as the matrix for the aroma reconstruction experiments.

### 2.3 Extraction of volatile compounds

Weigh 10 g of flue-cured tobacco leaf sample. Crush the tobacco into coarse particles and place them in a conical flask. Add 100 mL of double-distilled dichloromethane as the extraction solvent, along with 50 μL of deuterated n-hexanol (D13) at a concentration of 0.948 mg/mL as the internal standard (Jiang et al., 2024). Seal the conical flask and extract continuously for 0.5 h at room temperature using a constant-temperature magnetic stirrer (rotation speed 1000 r/min, DF-101S, Gongyi Yuhua Instrument Co., Ltd.). Following extraction, the mixture was filtered, and the residue was retained. The filtrate was supplemented with another 100 mL of distilled dichloromethane, and the extraction–filtration process was repeated twice. The three combined extracts were transferred to a solvent-assisted distillation apparatus, wherein the volatile components were subjected to phase separation under ultra-high vacuum (10^−5^ mbar). The obtained fraction was then dried over anhydrous sodium sulfate and subsequently concentrated to 1.5 mL using a Vigreux column (50 cm × 1 cm). Impurities were removed by filtration through a microporous membrane, and the solution was further concentrated to 1 mL using nitrogen purging. The sample was sealed and stored in a -40 °C ultra-low temperature freezer for subsequent analysis (Wu et al., 2026b).

### 2.4 GC-O-MS analysis

The pretreated samples were analyzed using gas chromatography–mass spectrometry (GC-MS) (Agilent 7820-5977, Agilent Technologies, USA) combined with olfactory detection (GC-O) to separate and identify volatile compounds, under the following conditions: A TG-WAX capillary column (30 m × 0.25 mm × 0.25 μm, Thermofisher, USA) was used to separate the volatile components. A four-way split system simultaneously connected the mass spectrometer detector and the olfactory detection port to enable simultaneous component identification and aroma perception recording. Olfactory detection was performed by three professionally trained sensory evaluators, each of whom independently smelled the samples and recorded the perceived aroma characteristics (Wu et al., 2026b).

GC analysis employed a temperature gradient starting from 50°C (3 min), then sequentially rising to 90°C at 5°C/min (6 min), 100°C at 4°C/min (5 min), 140°C at 4°C/min (8 min), and finally 230°C at 6°C/min (5 min), with ultra−high−purity helium (99.99%) as the carrier gas at a constant flow rate of 1.0 mL/min and an injection volume of 2 μL.

MS conditions: Electron impact (EI) ionization was used with an ionization energy of 70 eV and an ion source temperature of 250 °C. The mass scan range was 40–350 m/z in full scan mode, with a solvent delay time set to 3.8 min (Wu et al., 2026a).

### 2.5 GC-MS analysis

A gas chromatography system coupled with a single quadrupole mass spectrometer was employed to establish a dedicated detection platform for volatile compounds in flue-cured tobacco leaves (Liu et al., 2026). The chromatographic conditions were identical to those used in the GC-O-MS analysis described in Section 2.4; both the column temperature profile and mass spectrometry detection conditions were consistent with those in “2.4 GC-O-MS Analysis” to ensure uniformity of testing conditions and comparability of experimental data (Zhong et al., 2025).

### 2.6 AEDA analysis

Using the Aroma Extract Dilution Analysis (AEDA) method, the intensity of characteristic aroma compounds in flue-cured tobacco leaves was measured via a GC-O-MS detection platform. The Flavor Dilution (FD) factor was calculated to screen for and identify key aroma-active compounds in flue-cured tobacco leaves (Schulze et al., 2024).

Using dichloromethane as the diluent, the flavor-enriched extract of flue-cured tobacco leaves is serially diluted in geometric progression (1:2^^^n, where n represents the dilution order), and each dilution is subject to gas chromatography–olfaction (GC-O) until no odor is detected in three consecutive assessments; The maximum dilution factor at which the aroma is still detectable is defined as the flavor dilution (FD) factor.

To eliminate the influence of individual differences in olfactory sensitivity on the experimental results and ensure the accuracy and reliability of the data, each dilution series sample is independently evaluated by three professionally trained sensory evaluators (one male and two females). A dilution series is considered valid only when all three evaluators simultaneously perceive the target aroma; the corresponding dilution level is then recorded and used for FD value calculation.

### 2.7 Qualitative identification of aroma compounds

Four methods were used for cross-validation in the qualitative identification of aromatic compounds to ensure the accuracy and reliability of the results (Li et al., 2013). (i) Mass spectral library matching: The acquired mass spectra were searched against the NIST 14 library using the instrument’s software to obtain preliminary identifications;(ii) Retention index (RI) comparison: Linear retention indices on the TG-WAX column were calculated for each volatile component and compared with literature and authoritative database values;(iii) Olfactometric matching: The odor characteristics of the detected compounds were compared with those of authentic standards to confirm consistency;(iv)Standard confirmation: Under identical chromatographic conditions, reference standards were injected; the final identification was confirmed by matching retention times, characteristic ions, and relative ion abundances.

### 2.8 Quantitative analysis by internal standard calibration

The internal standard calibration curve method was used to perform precise quantitative analysis of key aroma-active compounds (FD ≥ 4) identified by AEDA screening (Wang H et al., 2024). Standard solutions of varying concentrations were prepared by dissolving the standards in dichloromethane. The mixtures were then sequentially diluted to obtain seven different concentration gradients. Deuterated n-hexanol (D13) was added as an internal standard to ensure that the internal standard concentration was constant and equal to the theoretical concentration of the sample after concentration to 1 mL using nitrogen purging. Data were collected using the Selective Ion Monitoring (SIM) mode of GC-MS to obtain the peak areas of the standards and the internal standard at different concentrations. Finally, a calibration curve was constructed by plotting the ratio of the peak areas of the standards to the internal standard against their corresponding concentration ratios; see **Table 1** for details.

**Table 1:** Aromatic compound content.

Name	CAS Number	Standard curve	R²	Content (μg/g)				OAV			
				ZY-C	ZY-B	HB-C	TR-C	ZY-C	ZY-B	HB-C	TR-C
Hydroxyacetone	116-09-6	y = 0.5171x + 0.0606	0.9991	0.64±0.04	0.12±0.05	1.24±0.14	0.57±0.06	0.06	0.01	0.12	0.06
Acetic acid	64-19-7	y = 0.2684x + 0.0006	0.9999	457.64±33.90	247.26±28.90	376.59±59.45	347.39±1.92	4.62	2.50	3.80	3.51
Benzaldehyde	100-52-7	y = 0.5705x + 0.0133	0.9992	0.02±0.02	0.02±0.01	0.02±0.00	0.02±0.00	0.92	1.02	0.79	0.78
Linalool	78-70-6	y = 0.4913x + 0.0463	0.9997	0.10±0.00	0.06±0.02	0.10±0.05	0.12±0.01	16.88	9.60	16.47	20.64
2-Methyl butyric acid	116-53-0	y = 1.0621x - 0.0197	0.9999	0.14±0.01	0.10±0.00	0.20±0.02	0.14±0.01	0.07	0.05	0.09	0.06
N-Methyl-2-pyrrolidone	872-50-4	y = 0.5264x + 0.0551	0.9999	2.03±0.12	1.63±0.42	28.06±1.62	2.52±0.48	0.05	0.04	0.68	0.06
(E)-5-isopropyl-8-methylnona-6,8-dien-2-one	54868-48-3	y = 0.1463x + 0.0016	0.9996	0.30±0.05	0.36±0.16	0.99±0.14	0.21±0.01	0.92	1.11	3.10	0.65
Phenethyl alcohol	60-12-8	y = 0.978x + 0.1378	0.9999	0.84±0.07	3.99±2.08	2.32±0.47	6.25±3.55	1.51	7.12	4.14	11.16
7,11,15-trimethyl-3-methylidene-hexadec-1-ene	504-96-1	y = 0.3296x + 0.2474	0.9974	280.51±25.53	257.34±44.25	797.25±139.60	321.93±33.04	–	–	–	–
3-Hydroxy-2-methyl-4H-pyran-4-one	118-71-8	y = 0.3252x - 0.2097	0.9938	7.56±0.29	7.37±0.81	6.35±0.40	6.95±0.31	6.09	5.95	5.12	5.61
p-Anisaldehyde	123-11-5	y = 1.7874x + 0.0017	0.9999	0.03±0.03	0.01±0.00	0.02±0.00	2.27±0.70	1.25	0.31	0.85	1.13
D - (-)-PANTOLACTONE	599-04-2	y = 0.3605x + 0.0405	0.9999	0.30±0.03	0.08±0.01	2.95±0.22	131.88±6.68	0.00	0.00	0.01	3.77
p-Cresol	106-44-5	y = 0.8326x + 0.0493	0.9994	0.10±0.01	0.06±0.01	0.12±0.03	0.26±0.06	25.90	15.83	30.77	–
Methyl palmitate	112-39-0	y = 0.1972x + 0.0014	0.9999	1.85±0.26	3.95±1.06	4.25±1.50	0.84±0.02	0.92	1.98	2.13	1.87
2,3-Dihydro-3,5-dihydroxy-6-methyl-4(H)-pyran-4-one	28564-83-2	y = 0.1953x - 0.0144	0.9989	131.75±3.58	74.38±3.43	136.11±29.47	9.16±0.43	3.76	2.13	3.89	1.50
3-Hydroxy-2-methylpyridine	1121-25-1	y = 0.344x + 0.0033	0.9999	0.27±0.07	0.07±0.02	0.44±0.10	2.27±0.70	–	–	–	1.13
3-Hydroxypyridine	109-00-2	y = 0.2159x - 0.0253	0.9990	0.74±0.00	0.82±0.02	1.01±0.11	131.88±6.68	1.65	1.82	2.25	3.77
Phenylacetic acid	103-82-2	y = 0.4287x - 0.6974	0.9902	9.13±0.02	8.74±0.36	10.38±0.76	0.26±0.06	1.50	1.43	1.70	–
D-Methandienone	23726-93-4	–	–	0.00625	0.0125	0.00625	0.003125	–	–	–	–

### 2.9 Aroma activity value

The Odor Activity Value (OAV) is calculated using the following formula: OAV = Ci/OTi, where Ci is the concentration of the target aroma compound and OTi is the olfactory threshold of the target aroma compound. Threshold data were sourced from relevant monographs and existing literature (Liu, 2021); in this study, the odor thresholds of compounds in an aqueous system were selected as the basis for calculation. The Odor Activity Value (OAV) is a commonly used metric for evaluating the contribution of aromatic compounds. If OAV < 0.1, the compound’s contribution to the aroma is negligible; if OAV ≥ 0.1, the compound contributes to the aroma, with a higher value indicating a greater contribution. When OAV exceeds 1, it indicates that the compound’s concentration in the sample has reached or exceeded its odor threshold in water, thereby making a significant contribution to the overall aroma profile of the tobacco leaf. IBM SPSS Statistics software was used to perform analysis of variance (ANOVA) and t-tests, with P < 0.05 indicating statistical significance (Qin et al., 2026).

### 2.10 Sensory evaluation

Sensory evaluation was conducted in a dedicated sensory analysis laboratory maintained at 25 ± 1 °C. A panel of 18 professionally trained evaluators (6 males and 12 females, aged 20–30 years) with extensive sensory evaluation experience was recruited. All panelists were free of olfactory impairments (Wang L et al., 2024).

The sensory evaluation was conducted in two rounds. In the first round, each panelist assessed the olfactory characteristics of tobacco leaf samples (3 g per sample) independently and recorded all perceived odor descriptors. The recorded terms were statistically summarized, followed by a panel discussion that led to the identification of seven core descriptors: honey−sweet, roasted, hay, floral, sour, woody, and fruity.

In the second round, the intensity of each descriptor was rated on a 0–10 scale, defined as follows: 0 (no perception), 1–2 (threshold), 3–4 (weak), 5–6 (moderate), 7–8 (strong), and 9–10 (very strong). Each panelist performed three independent olfactory tests per sample, and the arithmetic mean of the three ratings was taken as the final intensity score. Radar charts were generated to visualize the aroma profiles (Shi et al., 2024).

### 2.11 Aroma reconstruction deficiency experiment

The reconstruction experiment verifies the contribution and irreplaceability of key aroma compounds to the overall aroma of the sample by constructing an aroma reconstruction model and combining it with missing compound tests. The specific procedure is as follows: in the re-composition experiments, key aroma compounds in flue-cured tobacco samples were screened based on their odor activity values (OAV ≥ 0.1). Where olfactory thresholds were unavailable for certain aroma compounds, the results of aroma extract dilution analysis (FD ≥ 4) were used as a supplementary screening criterion; Subsequently, the key aroma compounds are blended according to their actual quantitative concentrations in the original sample. The blended aroma mixture is added to odorless vials containing cellulose powder, equilibrated at room temperature, and prepared as an aroma reconstruction model. A comparative sensory evaluation was conducted between this reconstructed model and the original sample to measure the intensity of seven odor attributes. The average scores for each attribute were calculated and plotted on a flavor radar chart. The accuracy of the key aroma components was confirmed by assessing the degree to which the reconstructed model reproduced the aroma of the original sample. Deficiency Test: Conducted based on the aforementioned reconstruction model, this test involved sequentially and selectively omitting one key aroma compound at a time to prepare multiple single-compound deficiency models. The triangular test method was used to verify the key role of each compound: Three brown glass bottles (containing two complete reconstruction models and one single-compound-deficient model) were presented in random order to 18 professionally trained sensory evaluators. The evaluators were asked to identify samples with different aromas based on overall aroma characteristics. Test results were expressed as significant differences, with the following criteria: Extremely significant difference (number of correct identifications ≥ 13, α ≤ 0.001), highly significant difference (number of correct identifications ≥ 12, α ≤ 0.01), significant difference (number of correct identifications ≥ 10, α ≤ 0.05). These results further validate the accuracy and irreplaceability of the key aroma-active compounds in the samples.

### 2.12 Data analysis

All experiments were conducted using three independent biological replicates per sample (n = 3). One-way analysis of variance (ANOVA) was performed, followed by Tukey’s HSD *post-hoc* test (p < 0.05), to compare the concentrations and OAV values among the four samples. Sensory evaluation was carried out by 18 assessors, with each assessor conducting three independent evaluations. For AEDA, a dilution series was considered valid only if all three assessors detected the same aroma. The results of the triad test were evaluated against binomial critical values. For the data visualization analysis, Origin 2026 professional graphing software was used to present the relevant data: radar charts (spider charts) were employed to visualize the sensory attributes based on the sensory evaluation scores (Shi et al., 2024).

## 3 Results and discussion

### 3.1 Qualitative identification of aroma compounds by GC-O-MS

To investigate the aroma-active compounds in flue-cured tobacco leaves and compare the differences in aroma compounds among these four varieties of flue-cured tobacco, GC-O-MS analysis was employed. This analytical method combines the separation capabilities of gas chromatography for volatile compounds with human olfactory discrimination. **Table 2** details the results of the GC-O analysis of the aroma compounds in tobacco leaves.

**Table 2:** Olfactory evaluation results for the four origins.

Number	Name	CAS number	Dilution ratio				Fragrance	Qualitative methods	RI
			ZY-C	TR-C	HB-C	ZY-B			
1	2-Methyl-3-buten-2-ol	115-18-4	2	1	1	1	earthy	MS/RI/O/STD	1020
2	Unknown		4	8	4	4	Pungent odor	O	1080
3	Hydroxyacetone	116-09-6	4	4	1	1	Fresh, sweet aroma	MS/RI/O/STD	1300
4	Acetic acid	64-19-7	128	64	32	64	tangy	MS/RI/O/STD	1427
5	Benzaldehyde	100-52-7	8	4	2	1	Almond scent	MS/RI/O/STD	1523
6	Linalool	78-70-6	8	2	1	16	The aroma of Sichuan pepper	MS/RI/O/STD	1541
7	2-Methyl butyric acid	116-53-0	8	8	8	8	Sour cream aroma	MS/RI/O/STD	1661
8	N-Methyl-2-pyrrolidone	872-50-4	8	8	8	64	smell of ammonia	MS/RI/O/STD	1674
9	(E)-5-isopropyl-8-methylnona-6,8-dien-2-one	54868-48-3	16	32	8	128	Carrot, tea aroma	MS/RI/O/STD	1713
10	Unknown		256	128	256	512	sour and musty odor	O	1831
11	Phenethyl alcohol	60-12-8	256	128	256	128	rose scent	MS/RI/O/STD	1934
12	7,11,15-trimethyl-3-methylidene-hexadec-1-ene	504-96-1	16	8	2	8	green	MS/RI/O/STD	1936
13	3-Hydroxy-2-methyl-4H-pyran-4-one	118-71-8	16	32	4	8	Caramel-sweet aroma	MS/RI/O/STD	1966
14	p-Anisaldehyde	123-11-5	16	–	–	8	Sweet aroma	MS/RI/O/STD	2023
15	Unknown		16	64	4	1	Sweet floral scent	O	2032
16	D-(-)-PANTOLACTONE	599-04-2	64	64	32	64	Caramelized sweetness	MS/RI/O/STD	2035
17	Unknown		256	128	256	128	tangy	O	2052
18	Unknown		16	8	2	8	Sweet aroma	O	2079
19	p-Cresol	106-44-5	32	16	16	8	phenolic odor	MS/RI/O/STD	2085
20	4,7,9-Megastigmatrien-3-one #1	38818-55-2	16	16	16	16	the scent of tobacco	MS/RI/O	2149
21	4,7,9-Megastigmatrien-3-one #2	38818-55-2	16	8	128	1	the scent of tobacco	MS/RI/O	2191
22	Unknown		64	64	16	128	Soy sauce aroma	O	2200
23	Methyl palmitate	112-39-0	4	8	2	16	Mineral wax fragrance	MS/RI/O/STD	2214

MS indicates identification based on the NIST spectral library; RI indicates identification based on retention index; O indicates identification based on aromatic characteristics; STD indicates identification based on a standard sample.

**Figure 1:**
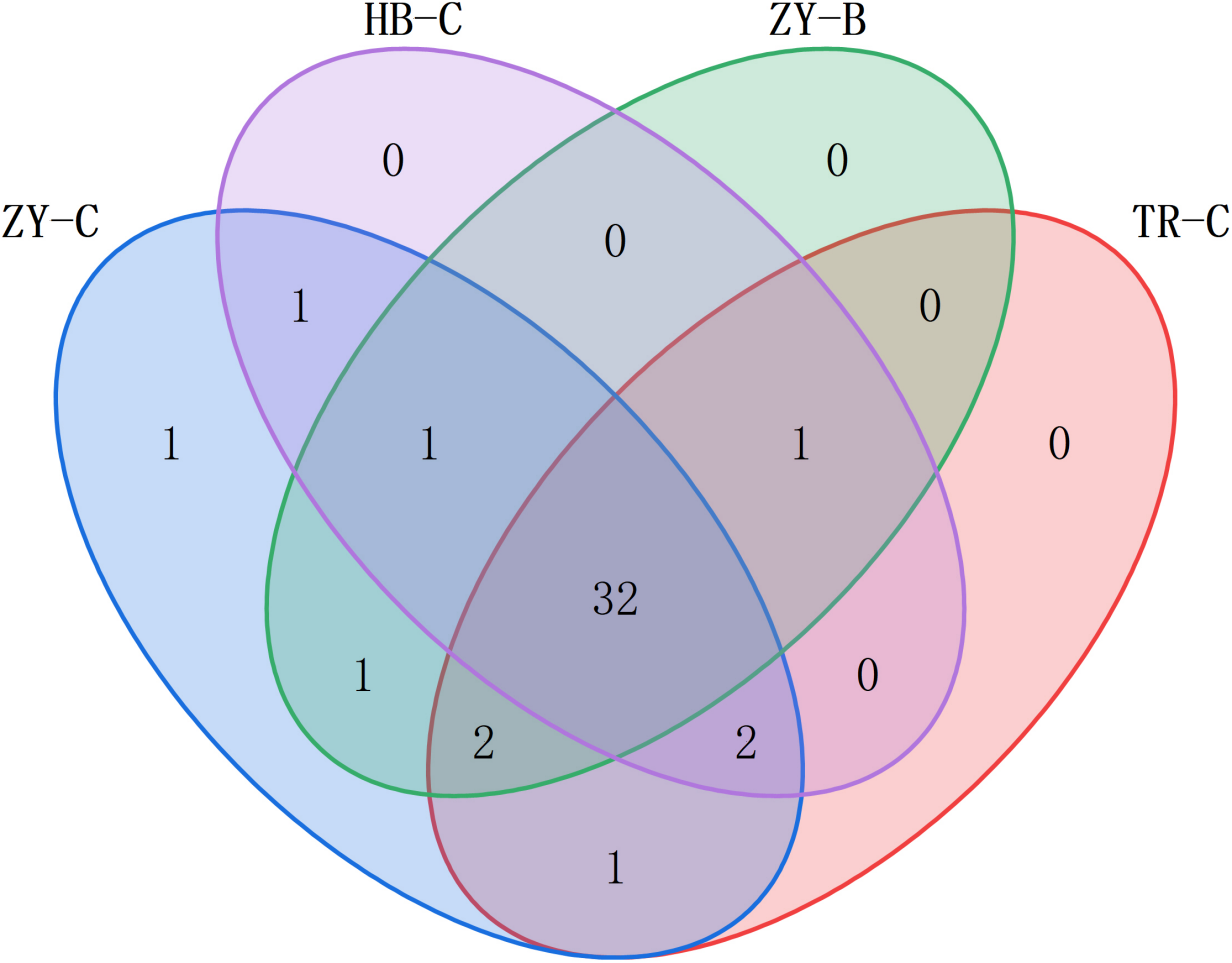
Venn diagram of aromatic compounds in four types of flue-cured tobacco leaves.

As shown in the **Figure 1**, a total of 42 aroma compounds were detected in the four types of flue-cured tobacco leaves. Specifically, 22 compounds were identified in the ZY-C standard sample; 22 in the TR-C standard sample; 22 in the HB-C standard sample; and 23 in the ZY-B standard sample. The quantitative concentrations of these identified compounds are presented in **Table 1**.

There was little variation in the types of compounds detected by olfactory analysis across the four samples; after qualitative analysis of the reference standards, only two distinct compounds were identified: p-methoxybenzaldehyde (anise aldehyde, with a green and anise-like aroma) and 2,3’-bipyridine (with a tobacco-like aroma and a structure similar to nicotine). The former was not detected in TR-C and HB-C, while the latter was not detected in ZY-C; these distinct compounds may be the key factors responsible for the differences in the honey-sweet aroma characteristics of the tobacco leaves. Since β-Damascenone showed no peaks in the chromatogram and lacked effective mass spectrometry fragments, three candidate compounds with similar odors and close retention indices were selected through olfactory screening, and their identities were ultimately confirmed by comparison with standard samples. All 23 compounds identified are previously reported; at trace levels (μg/g), they are generally recognized as safe with no known toxicity, and many also occur in other Solanaceae plants, supporting chemotaxonomic consistency.

The 23 compounds identified from the standard included: 8 ketones (the highest proportion, primarily contributing sweet aromas, supplemented by grassy and fruity notes), 3 heterocyclic compounds, 3 alcohols, 3 acids, 2 aldehydes, 2 phenols, 1 alkene, and 1 ester. TR-C and HB-C each lacked one aldehyde, ZY-C lacked one heterocyclic compound, while ZY-B identified all 23 compounds. Among these, β-Damascenone and megastigmatrienone are characteristic tobacco aroma compounds that form the material basis of the typical aroma of flue-cured tobacco; green-scented compounds such as linalool, phenethyl alcohol, and benzaldehyde enrich the aroma profile; Neophytadiene serves as both a characteristic tobacco aroma component and an aroma precursor, contributing directly and indirectly to the aroma quality of flue-cured tobacco (Liu et al., 2023). No distinct roasted aroma compounds were detected by olfactory analysis, indicating a discrepancy with the overall aroma profile of flue-cured tobacco; this represents an important direction for future research (Mao et al., 2020).

### 3.2 Screening of key aroma compounds by AEDA

To identify key aroma compounds in flue-cured tobacco leaves, AEDA analysis was performed. AEDA identifies the compounds that contribute most significantly to the overall flavor through stepwise dilution. Among the ZY-C, TR-C, HB-C, and ZY-B samples, there were 32, 26, 17, and 22 compounds with FD ≥ 4, respectively. However, the number of compounds identified by standard samples and mass spectrometry was 23, consistent with the results in the previous section. These findings further demonstrate the diversity of aroma compounds in the four flue-cured tobacco leaf samples and their unique flavor characteristics.

**Figure 2:**
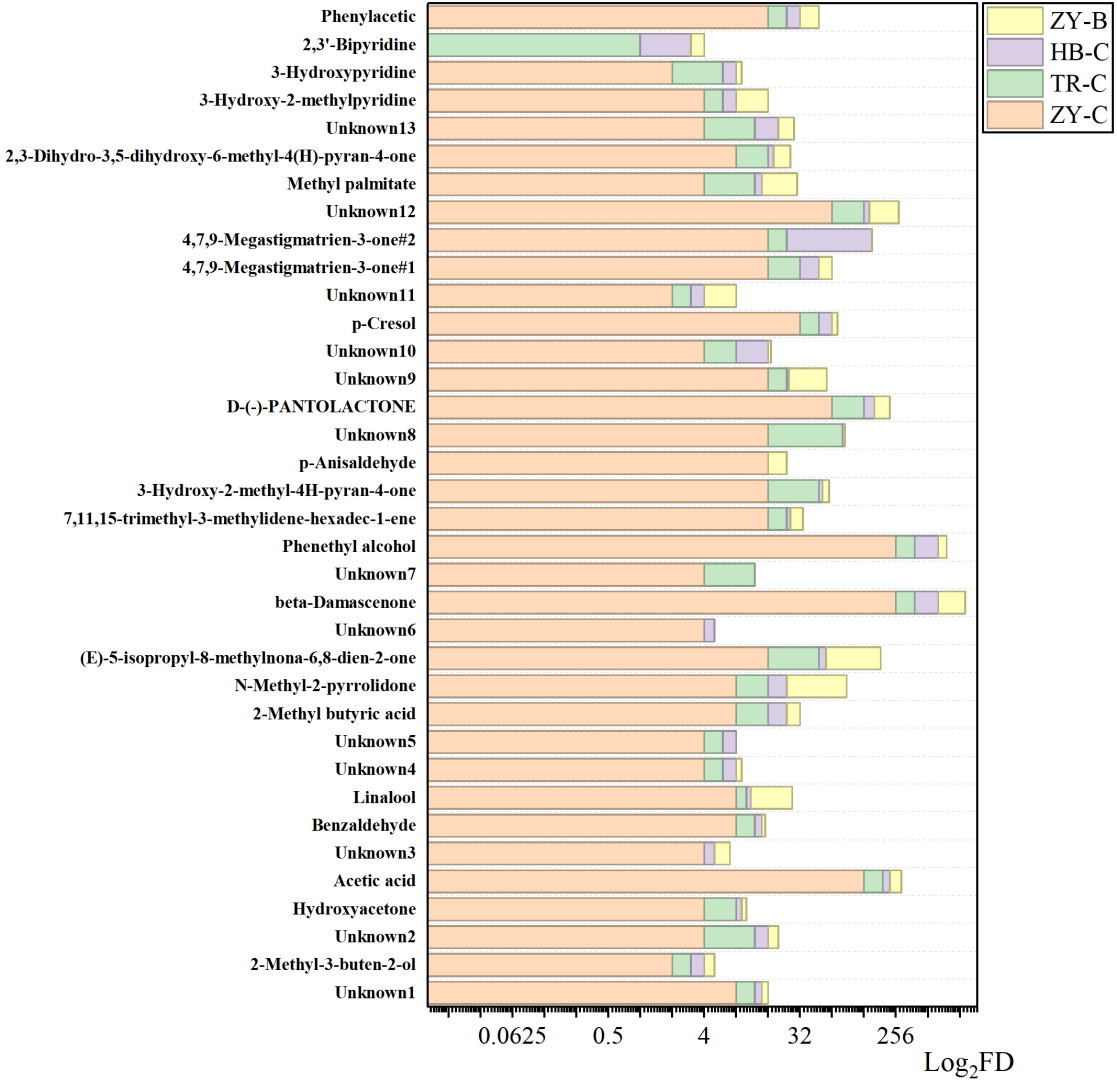
FD factors of aroma-activating compounds in four types of tobacco leaves.

From **Figure 2**, we can see that, β-Damascenone (FD = 128–512) and phenethyl alcohol (FD = 128–256) are highly active aromatic components commonly found in all four samples, imparting rose/fruity and green-sweet notes, respectively, and synergistically enhancing the subtlety of the tobacco aroma (Wang et al., 2015). The aromatic differences among the samples are primarily determined by the FD values (concentrations) of the compounds, rather than by differences in compound types. The following figures all represent the FD factor. In ZY-B, β-Damascenone exhibited the highest FD value (512), with significant contributions from phenethyl alcohol (128), solanone (128, a marker of tobacco-like aroma), acetic acid (64), linalool (16), and p-methoxybenzaldehyde (8), resulting in a fresh and mellow aroma. In ZY-C, acetic acid (128) and phenylacetic acid (16) provide an acidic aroma, while furanone (64) and maltol (16) present a caramel-like sweetness, and megastigmatrienone (16) supports the tobacco character.

TR-C and HB-C share similar aroma profiles, but their FD values differ significantly: TR-C is dominated by β-Damascenone, phenethyl alcohol, furanone, and acetic acid (64–128); in HB-C, β-Damascenone, phenethyl alcohol (both 256), and megastigmatrienone (128) are more prominent (Yang et al., 2025).

(E)-5-isopropyl-8-methylnona-6,8-dien-2-one (8–128) was higher in TR-C and ZY-B; furanone (32–64) was prominent in ZY-C and TR-C; maltol (4–32) synergistically enhanced the sweet and mellow sensation; acetic acid (32–128) mitigated pungency, with the highest levels in ZY-C; Linalool (1–16) was highest in ZY-B, imparting a fresh aroma; p-cresol (8–32) was higher in ZY-C, enriching the aromatic complexity. These differences collectively constitute the characteristic aroma profiles of each sample.

Although the compositions of aroma-active compounds in the four flue-cured tobacco leaf samples are similar, they exhibit diversity and specificity. The ZY-C sample contains the highest number of key aroma-active compounds (FD≥4) (32 types), featuring the richest aroma composition and the most harmonious layers; The ZY-B sample contains a large number of highly active aroma compounds (FD ≥ 64), centered on β-Damascenone and linalool; the TR-C sample has a moderate number of aroma compounds, with aromas leaning toward a caramel-sweet and herbal tobacco profile; the HB-C sample has the fewest important aroma compounds (17 types), and its aroma complexity and intensity are relatively low.

### 3.3 Quantitative analysis of compounds and OAV analysis

To further clarify the differences in aroma quality among flue-cured tobacco leaves of different grades and origins, we conducted quantitative analysis using GC-MS to determine the concentrations of 18 known volatile aroma compounds in ZY-C, ZY-B, TR-C, HB-C, using GC-MS quantitative analysis. The standard curves exhibited good linearity (R² ≥ 0.99). Additionally, the actual contribution of these compounds to the aroma of the tobacco leaves was evaluated using the Odor Activity Value (OAV) (Zhu et al., 2017). The standard curves, concentrations, and OAV values are presented in **Table 1** respectively.

Overall, there are significant variations in compound concentrations across different grades and origins, exhibiting a distribution pattern characterized by “high concentrations dominated by organic acids and terpenes, while medium-to-low concentrations are dominated by alcohols, aldehydes, ketones, and pyridines.” The following discussion focuses on compounds with concentrations ≥1 μg/g.

**Figure 3:**
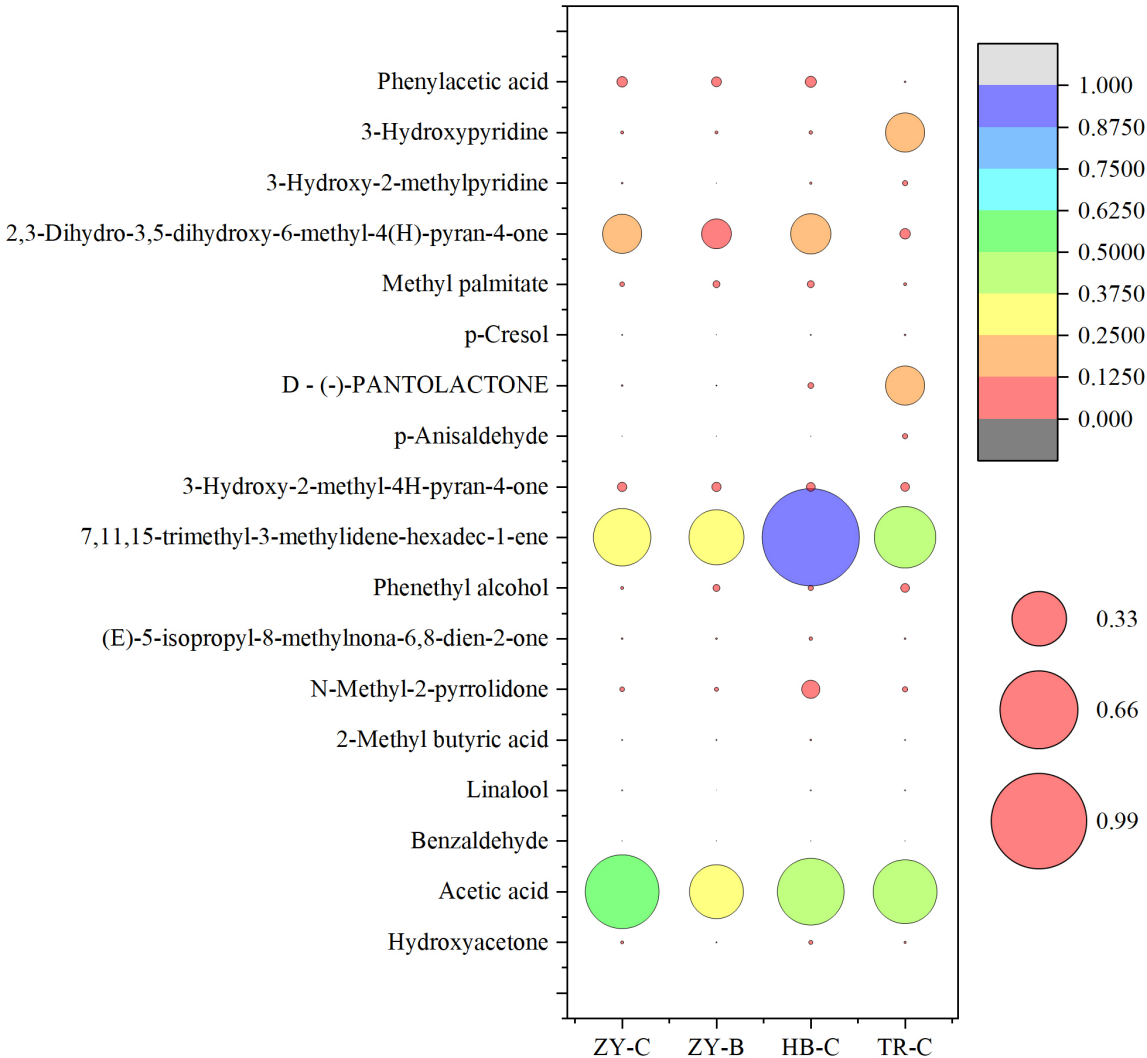
Bubble chart showing the content of key aromatic compounds in different tobacco varieties.

As can be seen from **Figure 3**, high-content compounds are key components of the aromatic profile of tobacco leaves, primarily including acetic acid, neophytadiene, and DDMP (2,3-dihydro-3,5-dihydroxy-6-methyl-4H-pyran-4-one). Significant differences were observed in neophytadiene content: HB-C had the highest value (797.25 μg/g), significantly higher than the other samples; TR-C was next (321.93 μg/g); ZY-C (280.51 μg/g) and ZY-B (257.34 μg/g) were similar, indicating that the Hubei and Tongren production areas have an advantage in the accumulation of terpenoid precursors (Ting et al., 2021). Acetic acid, as an organic acid compound, can regulate the acidity of the aroma and mitigate its pungency (Jiang et al., 2021). The content ranking was: ZY-C (457.64 μg/g) > HB-C (376.59 μg/g) > TR-C (347.39 μg/g) > ZY-B (247.26 μg/g). Within the same origin, Grade C was significantly higher than Grade B, which may be related to maturity and leaf position. Furanones, which impart a caramel-like sweetness, showed the following content rankings: HB-C (136.11 μg/g) ≈ ZY-C (131.75 μg/g) ≈ TR-C (131.88 μg/g) > ZY-B (74.38 μg/g), further confirming the influence of grade on aroma accumulation, while no significant differences were observed among Grade C samples. Maltol (caramel-sweet aroma) content was higher in samples from the Zunyi region: ZY-C (7.56 μg/g) > ZY-B (7.37 μg/g) > TR-C (6.95 μg/g) > HB-C (6.35 μg/g), demonstrating the influence of origin on sweet-aroma compounds. Phenylacetic acid (floral aroma) was highest in HB-C (10.38 μg/g), with TR-C and ZY-C being similar, and Grade C slightly higher than Grade B. Phenethyl alcohol (mild floral aroma) was highest in TR-C (6.25 μg/g), significantly higher than in other samples (Zhang et al., 2025). Compounds with low concentrations should be analyzed in conjunction with OAV analysis, which helps identify aroma compounds with high thresholds, low concentrations, but significant contributions (Wang et al., 2015).

**Figure 4:**
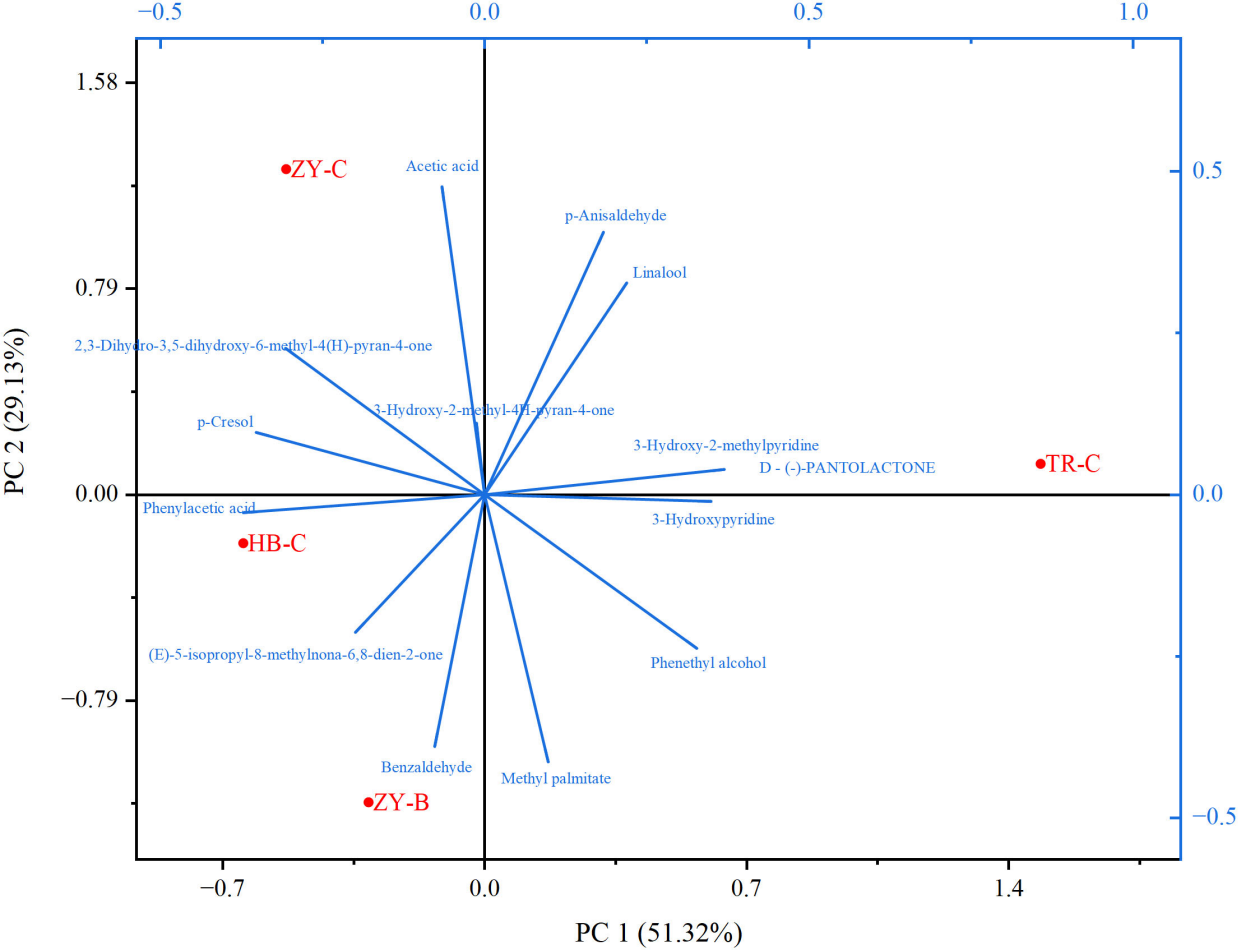
PCA analysis based on four key components of tobacco leaves.

The key aroma-active compounds with OAV ≥ 1 in the four samples were as follows: 12 in ZY-C, 10 in ZY-B, 12 in TR-C, and 11 in HB-C. Overall, this reflects the characteristic that “differences between origins are greater than differences within the same origin grade (Fang et al., 2024).” Differences in core aroma contributions among samples: In ZY-C, the compounds with higher OAV values were, in descending order, p-cresol (25.90), linalool (16.88), maltol (6.09), and acetic acid (4.62). Although p-cresol is present at a low concentration (0.10 μg/g), its extremely low odor threshold imparts a strong smoky and phenolic character to the tobacco leaves; linalool, as a typical floral compound, contributes a delicate floral undertone to ZY-C; maltol and acetic acid contribute caramel-like sweetness and acidic notes, respectively. These four high-OAV compounds collectively constitute the complex aromatic profile of ZY-C (Yang et al., 2019). In ZY-B, aroma compounds with OAV ≥ 1 include p-cresol (15.83), linalool (9.60), phenethyl alcohol (7.12), maltol (5.95), and acetic acid (2.50) (Qin et al., 2026). It is worth noting that the OAV value of phenethyl alcohol in ZY-B (7.12) is significantly higher than that in ZY-C (1.51). Although its absolute content (3.99 μg/g) is not particularly high, due to its low aroma threshold (approximately 0.06 μg/g), it makes a significant contribution to the rose-like aroma profile of ZY-B. Furthermore, p-cresol was the only compound with an OAV greater than 10 in ZY-B, whereas ZY-C contained two (p-cresol and linalool), suggesting that Grade C tobacco leaves may possess a stronger characteristic aroma intensity. HB-C contains no compounds with OAV > 35; it is dominated by p-cresol (OAV = 30.77) and acetic acid (OAV = 3.80), with a weaker contribution from phenethyl alcohol, resulting in a more pronounced tobacco-like aroma. Compounds such as benzaldehyde, solanone, and methyl hexadecanoate have OAV values slightly greater than 1 in some samples, serving to modify the overall aroma. Although dihydro-3-hydroxy-4,4-dimethyl-2(3H)-furanone is present in significant amounts in some samples, its threshold is extremely high and its OAV approaches 0, indicating that it makes little direct contribution to the aroma. These OAV-based compositional differences among the three samples are further illustrated by **Figure 4**.

The overall aroma profiles of TR-C and HB-C are more similar; there are 10 key aroma compounds common to both types of tobacco leaves, namely phenethyl alcohol, maltol, acetic acid, furanone, p-cresol, linalool, benzaldehyde, solanone, 3-hydroxypyridine, methyl hexadecanoate. These may represent relatively important common aroma compounds in Grade C flue-cured tobacco from different origins, collectively constituting the key substances of the tobacco’s base aroma; whereas ZY-C and ZY-B, as samples of different grades from the same origin, share 9 key aroma compounds, with only p-methoxybenzaldehyde exhibiting significant aroma activity in ZY-C (OAV = 1.25), while it made no significant contribution in ZY-B. This also reflects the differences in aroma compounds between tobacco leaves of different grades from the same origin.

B-Damascenone, the compound with the highest FD value among all samples, was identified based on olfactory analysis, refractive index (RI), and comparison with a standard. Consequently, it did not exhibit a peak area in the chromatogram, making it impossible to quantify using an internal standard calibration curve. To determine its approximate concentration and provide a rough estimate of the concentration of this key aromatic compound for subsequent recombinant deletion experiments, a 1 μg/g solution of damaskon was prepared according to Reference, a 1 μg/g β-Damascenone solution was prepared. Using the same dilution analysis method, the standard solution was sniffed until no odor was detectable, and the dilution factor was recorded. This factor was compared with the dilution factor in the sample. By applying the relationship between the dilution factor ratio and the concentration ratio, the concentration of β-Damascenone was determined.

### 3.4 Results of sensory evaluation and reorganization deficit experiments

**Figure 5:**
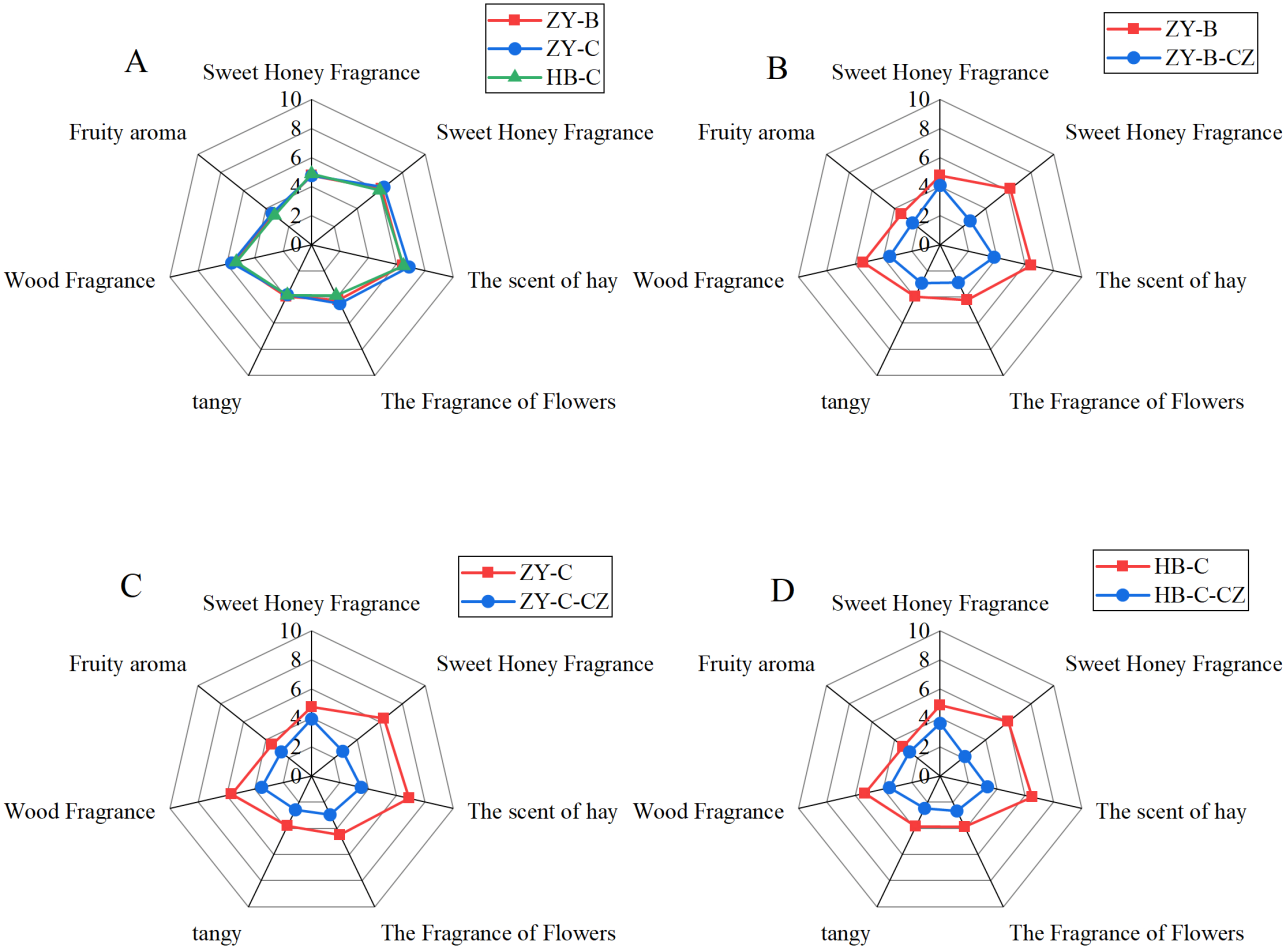
Sensory evaluation of aroma profiles from different origins and the corresponding reconstruction model. **(A)** Aroma profiles of the three tobacco leaf varieties **(B)** Comparison of the aroma profile of ZY-B with the reconstructed aroma profile **(C)** Comparison of the aroma profile of ZY-C with the reconstructed aroma profile **(D)** Comparison of the aroma profile of HB-C with the reconstructed aroma profile.

Based on the sensory evaluation and aroma profile analysis shown in **Figure 5**, the three tobacco samples (ZY-C, ZY-B, and HB-C) were compared across seven descriptors. Hay, roasted, and woody aromas dominated, exhibiting the highest average intensity (Qiao et al., 2017).

ZY-C exhibited the most complex aroma profile, with hay, woody, roasted, and floral notes ranking highest in intensity, while sour and fruity notes were relatively mild. ZY-B ranked in the middle overall; its sour and fruity note intensities were similar to those of ZY-C, but the intensities of hay, roasted, and floral notes were lower. HB-C exhibited the weakest overall aroma, with the lowest scores for floral, woody, and fruity attributes.

Significant differences were observed in hay, woody, and toasted aromas: ZY-C > ZY-B > HB-C. For floral aromas, ZY-C was the strongest, followed by ZY-B, and then HB-C. There was no significant difference in fruitiness between ZY-C and ZY-B, and sample distinguishability was low. HB-C exhibited a slightly higher honey-like sweetness, which may be related to its neofuran content, though this did not reach statistical significance. The ranking of acidity was ZY-B > HB-C > ZY-C, which was inconsistent with acetic acid content (ZY-C had the highest), suggesting that the perception of acidity is modulated by other aromatic compounds.

As can be seen from **Figure 5**, the results of the aroma reconstruction experiments for the four samples are as follows: ZY-C-CZ is similar to the original sample in terms of fruity and honey-like aromas, but the roasted, hay, and woody notes are significantly weaker. These three aromas are the core characteristics of ZY-C, and their absence may affect the overall harmony; appropriately increasing the proportion of roasted-related compounds such as maltol and p-cresol may improve the reconstruction accuracy (Yang et al., 2021). ZY-B-CZ exhibits generally low aroma intensity, with particularly insufficient roasted, hay, and woody notes. This may be due to the fact that only key compounds with OAV ≥ 1 were included during the reconstruction, while certain modifying pyrazine and phenolic derivatives (with OAV < 1 or not accurately quantified) were excluded. Additionally, the absence of reference standards such as megadienone led to insufficient perception of the relevant aromas. The HB-C-CZ sample exhibited poor reproduction of honey-like sweetness, with roasted and hay-like aromas also lower than those of the original sample. This is presumed to be related to the exclusion of sweet-enhancing compounds such as lactones and furanones, as well as their synergistic effects. Additionally, the contribution of the high content of neoflavanone and its degradation products remains to be investigated. Overall, the aroma profiles of the reconstituted samples were generally less rich than those of the original samples, which is closely related to the inability to accurately identify some compounds with high FD values during olfactory analysis. Improving the qualitative and quantitative analysis of key trace components to expand the aroma profile is a key focus for future research (Wang et al., 2021).

The knockout experiments, building on the recombination experiments, clarify the importance of each compound to the whole system, analyzing the role of each compound from the perspective of its presence or absence. The results are shown in **Table 3**.

**Table 3:** Results of the absence of key aromatic compounds in the three origin regions.

Number	Name	Significance		
		ZY-C	ZY-B	HB-C
1	Acetic acid	***	***	***
2	7,11,15-trimethyl-3-methylidene-hexadec-1-ene	*	***	–
3	2,3-Dihydro-3,5-dihydroxy-6-methyl-4(H)-pyran-4-one	–	–	–
4	Phenylacetic acid	*	–	**
5	3-Hydroxy-2-methyl-4H-pyran-4-one	–	–	*
6	Phenethyl alcoho	–	–	–
7	Methyl palmitate	–	–	–
8	3-Hydroxypyridine	–	–	–
9	(E)-5-isopropyl-8-methylnona-6,8-dien-2-one	–	–	–
10	3-Hydroxy-2-methylpyridine	–	–	–
11	Benzaldehyde	–	–	–
12	Linalool	–	–	–
13	p-Anisaldehyde	–	–	–
14	p-Cresol	–	–	–
15	beta-Damascenone	*	*	*

*, significant (α ≤ 0.05); **, highly significant (α ≤ 0.01); ***, extremely significant (α ≤ 0.001); -, not significant.

Among the 15 missing aroma compounds, acetic acid (α ≤ 0.001) was the only compound that showed extremely significant differences in all three samples. Quantitative results indicated that ZY-C had the highest acetic acid content, while HB-C had an intermediate level; however, this did not correlate linearly with the sensory ratings for sourness. This result is primarily attributed to perceptual interactions between aroma compounds within the complex tobacco matrix. Although acetic acid is the primary source of sourness, its perceived intensity is not determined solely by its concentration. Other volatile compounds may exert masking or synergistic effects on the perception of sourness. B-Damascenone (α ≤ 0.05) showed significant differences across all three samples. Although its absolute content was low, it made a significant contribution to floral and fruity aromas due to its extremely low odor threshold; this difference may be intrinsically linked to variations in the sensory ratings of floral and fruity aromas among the samples. HB-C had a slightly higher honey-sweet aroma score but showed no significant difference compared to the other samples; however, its maltol (α ≤ 0.05) content differed significantly, suggesting that the caramel-sweet aroma provided by maltol may evoke a sensory perception similar to honey-sweet aroma (Wu et al., 2024). In ZY-C, the models lacking neoflavanone (α ≤ 0.05) and phenylacetic acid (α ≤ 0.05) were also significant, with neoflavanone being significant at the α ≤ 0.01 level. Overall, only five substances (one-third of the total) showed significance, indicating that the lack of a rich aroma profile may be related to the qualitative absence of certain compounds.

## 4 Conclusion

This study employed molecular sensory science methods, including AEDA, GC-MS-O, OAV calculations, sensory evaluation and reconstruction-depletion experiments, to systematically characterize the aroma-active compounds in Guizhou honey-type flue-cured tobacco. A total of 42 aroma-active compounds were detected across the four tobacco leaf samples, of which 23 have been definitively identified. The study found that β-damascenone and phenethyl alcohol constituted the common, highly active core components across all samples. Among the samples, ZY-C exhibited the richest composition of active components, with 12 key compounds having OAV values ≥1, and its sensory characteristics were also the most desirable. The aromatic differences between samples were primarily attributed to variations in compound concentrations and ratios, rather than differences in compound types. Reconstruction experiments successfully reproduced the fruity and floral notes, but failed to fully replicate the baked, hay and woody notes, highlighting the indispensable role of trace components (including compounds with OAV < 1 and unidentified trace aroma substances). Deficiency experiments confirmed that acetic acid, β-Damascenone, neoterpene, phenylacetic acid and maltol make significant contributions to the overall aroma.

However, this study has several limitations. Firstly, due to the absence of detectable peaks, β-Damascenone, the compound with the highest FD factor, could not be directly quantified by gas chromatography–mass spectrometry (GC-MS); its concentration was estimated indirectly, which may introduce uncertainty. Future studies should employ more sensitive analytical platforms, such as full-range two-dimensional gas chromatography–gas chromatography–mass spectrometry (GC×GC-MS), to enable direct quantification at trace levels. Secondly, the reconstruction of baked, hay and woody aroma notes was incomplete, indicating that the current reconstruction library lacks some important aroma compounds with OAV < 1 (or those not yet identified), and that synergistic or masking effects between compounds have not been fully captured. Future research should expand the aroma library by systematically incorporating those low-OAV compounds that were consistently detected, and by synthesizing or obtaining reference standards for candidate compounds that are currently unavailable. Thirdly, this study was limited to four samples from three geographical origins and two grades; therefore, the generalizability of its conclusions to other regions or grading systems remains to be verified. Expanding the sample size and combining this with multivariate statistical modeling will help to confirm the observed trends. Addressing these limitations will contribute to a more comprehensive and accurate understanding of the honey-sweet tobacco flavor system, and facilitate the precise reproduction and targeted modulation of its aromatic characteristics.

## Data Availability

The raw data supporting the conclusions of this article will be made available by the authors, without undue reservation.
